# Correlating micro/meso pore evolution and chemical structure variation in a mild thermal treatment of a subbituminite

**DOI:** 10.1039/c7ra13215h

**Published:** 2018-03-09

**Authors:** Shipei Xu, Zhennan Han, Rongcheng Wu, Jiguang Cheng, Guangwen Xu

**Affiliations:** State Key Laboratory of Multi-phase Complex Systems, Institute of Process Engineering, Chinese Academy of Sciences Beijing 100190 China rwu@ipe.ac.cn gwxu@ipe.ac.cn; University of Chinese Academy of Sciences Beijing 100049 China; Institute of Industrial Chemistry and Energy Technology, Shenyang University of Chemical Technology Shenyang 110142 China

## Abstract

This work investigates the evolution of micro/meso pores during a mild thermal treatment of subbituminous coal based on the observation of coal structure changes with the gradual detachment of organic matter from the coal. Pores in coal can be described as super-micropores (*d* < 1 nm), micropores (1 nm < *d* < 2 nm) and mesopores (2 nm < *d* < 50 nm). The decomposition of the carboxyl group at 200 °C decreases the super-micropore volume. A mild and sustained reaction takes place at 300 °C to gradually change the aromaticity and CH_2_/CH_3_ ratio of the treated coal. The amount of micropore structure sharply decreases in the early stages of heating, while the amount of mesopore structure continuously decreases during the whole process. A dramatic reaction takes place at 400 °C to sharply change the aromaticity and CH_2_/CH_3_ ratio of the treated coal, while the detachment of volatile compounds from the coal matrix caused an evident variation in the mesopore structure of the coal. The aromaticity and CH_2_/CH_3_ ratio of coal organics are found to correlate with the volumes of super-micropores and mesopores, respectively. The super-micropores are identified as comprising the inter-layer distance between stacks of aromatic rings, and mesopores are the spaces between macromolecular aromatic rings which are inter-connected *via* aliphatic chains.

## Abstract 2

The aromaticity and CH_2_/CH_3_ ratio of coal organics correlate with the volumes of super-micropores and mesopores, respectively.

## Graphical abstract

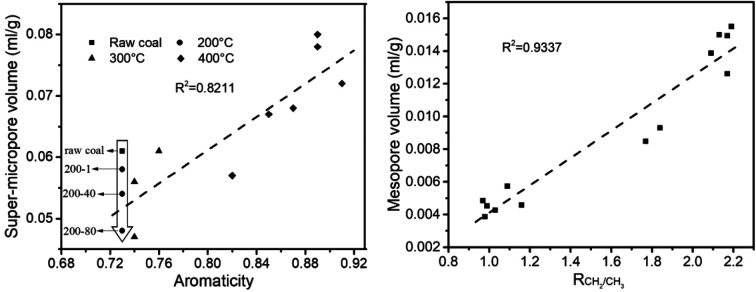

## 1. Introduction

Coal consists of complex organic species and inorganic species, which results in complex and heterogeneous structures.^1,2^ Understanding the structure of coal is crucially important for the efficient, clean, and value-added utilization of coal.^3^ Investigations into the physical and chemical structure of coal have always been the key subject of coal chemistry studies. The physical structure of coal mainly refers to the pore structure,^4^ and the chemical structure indicates fused aromatic moieties leading to a three-dimensional network of macromolecular coal structures. Thermal treatment of coal is an essential approach for understanding the chemical composition and molecular skeletal structure of coal.^5^ Meanwhile, the thermal treatment of coal is also of great significance for the efficient utilization of coal and for the study of the chemical composition of coal.^6–10^

Researchers have performed a lot of studies into the pore and chemical structures of coal during heat treatment.^11–14^ Several direct characterization tools are also widely used to obtain original structural information.^15–19^ Zhu *et al.*^20^ adopted Raman spectroscopy to characterize the carbon microstructure of char after thermal treatment and found that the char structure evolution behaved differently before and after 800 °C. Increasing the treatment temperature from 500 to 800 °C resulted in a significant decrease in the number of functional groups, a decrease in char yield, and an increase in the number of smaller pores. Structural defects and imperfections of carbon crystallites were gradually eliminated, and the poorly organized structure in the carbon materials gradually became ordered from 800 to 1200 °C. Feng *et al.*^21^ found smaller pores of coal samples further developed during the heat treatment above 400 °C, which was mainly due to an enhanced decomposition of surface groups and the release of volatile compounds. The stacking of carbon layers in a graphite-like structure happened during the heat treatment, which was associated with the development of a three-dimensional crystalline structure. These works have indeed studied the chemical and pore structure changes during thermal treatment. However, they mainly focused on the changes of coal at high temperatures at which the original structure of coal had been totally destroyed, making it hard to gain a good insight into the composition and structure of raw coal. There are very few studies investigating the gradual change of coal composition and structure at low temperatures and also for a long treatment time. Furthermore, the clarified relationship between the chemical and physical structures was mainly qualitative, thus requiring further studies for quantitative understanding.

In response to the findings above, this study aims to explore the relationship between the chemical and pore structure of a typical Chinese subbituminous coal under mild conditions. Under such gentle conditions, which are investigated for the first time, functional groups and small molecules gradually escaped from the coal to get slight and continuous changes in coal composition and structure. By investigating the changes, we could gain a deeper understanding of the complex structure in coal. We adopted CO_2_ and N_2_ gas adsorption to quantify pore structure changes and to provide a full-range distribution of micro/meso pore sizes. Obtaining two parameters, the aromaticity and ratio of CH_2_/CH_3_, by NMR was used to quantify the chemical structure change of coal. Subsequently, linear fitting was adopted to find out the relationship between the chemical and pore structure changes. This would promote our understanding of the chemical and pore structures of this type of coal in thermal conversion.

## 2. Experimental

A kind of subbituminous coal from Shenmu County (China) was used. It had a high volatile compound content of 34.47% (Table 1) and was crushed into 40–80 meshes for testing. Before the heat treatment, the coal was sealed and evacuated for 24 h at 80 °C (denoted as raw coal). The heat experiments were all performed in a quartz fixed bed reactor in a nitrogen atmosphere. The reactor had an internal diameter of 30 mm and a length of 400 mm. A sintered quartz plate at 200 mm above the reactor lower end supported the tested coal sample. The reactor was externally heated by an electric furnace, and the reaction temperature was controlled *via* a proportion integration differentiation controller interfaced with a thermocouple that measured the temperature just above the sintered plate. Nitrogen with a purity of 99.99% was used as the carrier gas and its flow was monitored using a mass flow meter. Thirty grams (30 g) of the sample in the center of the reactor was heated to a desired temperature in a N_2_ flow of 100 ml min^−1^. The nitrogen flow was stopped when the reactor temperature dropped to room temperature. Char was removed from the reactor and weighed after each experiment. Hereafter, a char sample “400-20” refers to the condition of heating at 400 °C for 20 hours. Table 1 summarizes the proximate and ultimate analysis data, which followed the ASTMD7582 method, for coal and chars.

**Table 1:** Proximate and ultimate analyses of coal sample

Sample	Proximate analysis (wt%, dry base)			Ultimate analysis (wt%, daf)				
	Volatile compounds	Ash	Fixed carbon	C	H	N	S	O^a^
Raw coal	34.47	4.60	60.93	80.88	5.19	1.20	0.71	12.02

a By difference.

Adsorption isotherms for N_2_ at −196 °C and for CO_2_ at 0 °C were measured in the Quantachrome Autosorb-6B/3B. The N_2_-adsorption isotherms were obtained for relative pressures (gas pressure against saturated vapor pressure, *P*/*P*_0_) of 0.001 to 0.995, and the CO_2_-adsorption isotherms were measured in a pressure range of 1 to 760 Torr. The samples were degassed under vacuum at 80 °C for 10 h before the adsorption measurements.^22^ On the basis of density functional theory (DFT), pore structure parameters were automatically calculated by computer software.^23^

All NMR spectra were recorded by single pulse excitation/magic angle spinning (SPE/MAS) on a Bruker AV300 spectrometer. The obtained spectra were further processed in Origin Pro 2016 using the Gaussian-curve function. The number of curves adopted is 16 and each one is characterized with its chemical shift at the peak and full width at half maximum of width (FWHM). Table 2 summarizes such parameters of peak assignment for the 16 curves.^24^

**Table 2:** Peak fitting parameters of ^13^C-NMR

Functional groups	Ketone, aldehyde	Carboxylic acid	Ar–O	Ar–C, H	R–O	–CH_2_	–CH_3_
Peak center (ppm)	202	187, 178	167, 153	140, 126, 113, 101	96, 76, 56	40, 31	20, 13
FWHM (ppm)	12–5	12–15	15–16	16–18	16–18	11–13	10–12

## 3. Results and discussion

### 3.1 Evolution of pore structure

In order to quantitatively analyze the pore structure in coal or char we adopted N_2_ and CO_2_ adsorption analyses. As we know, the coal and char samples containing very fine micropores are difficult to analyze at cryogenic temperatures because of their extremely slow diffusion at low relative pressures. Rodriguez-Reinoso *et al.*^25^ have reported that more than 100 h was required for measuring N_2_ adsorption isotherms at −196 °C of some carbons with fine pores approaching molecular sizes. Increasing the measurement temperature to −183 °C dramatically reduced the equilibration time and improved the accuracy of the obtained data. We thus just used N_2_ to analyze pores larger than 1 nm. Adsorption of CO_2_ at 0 °C has been widely applied to the analyses of carbon molecular sieves and microporous carbons.^26,27^ Pores with sizes smaller than 50 nm usually occupy more than 90% of the total surface area, thus greatly affecting gas adsorption and diffusion characteristics. Consequently, pores with diameters smaller than 50 nm are studied in detail by distinguishing them as super-micropores (*d* < 1 nm), micropores (1 nm < *d* < 2 nm) and mesopores (2 nm < *d* < 50 nm).

#### 3.1.1 Micropores and mesopores

**Fig. 1:**
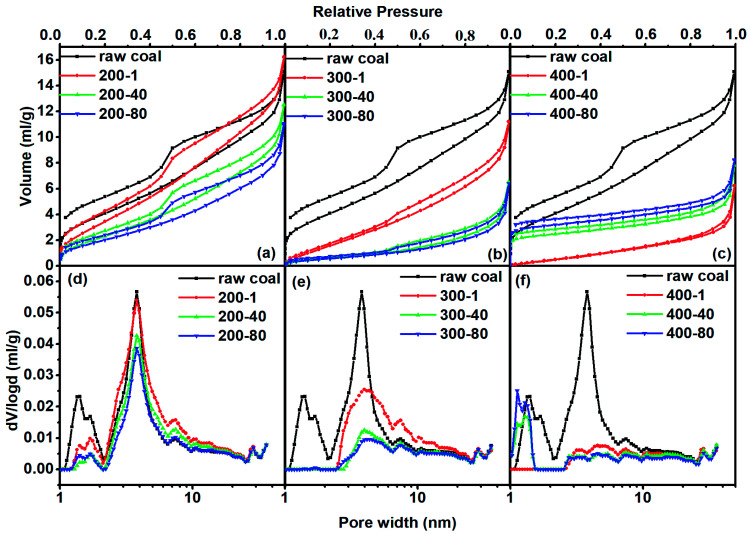
N_2_ adsorption isotherms [(a)–(c)] and the N_2_-DFT–PSD [(d)–(f)] for different samples.

Fig. 1 shows N_2_ adsorption isotherms (a–c) and DFT-pore size distributions (d–f) of samples from heat treatments at different temperatures and times. The pore size distribution (PSD) is fairly complex because all of the samples are micro–mesopore materials. The sorption behavior in micropores (pore width < 2 nm) is dominated by the interactions between fluid molecules and pore walls. In contrast, the sorption behavior in mesopores depends not only on the fluid–wall attraction but also on the attractive interactions among fluid molecules. These two parts of the PSD are always calculated by different methods.^28^ The method based on DFT allows us to analyze pore size accurately over the complete micro–mesopore size range. The PSD calculated from the adsorption isotherm matched well with the change of the bulge in the desorption line, indicating that this method is suitable for the samples.

For all samples their adsorption hysteresis loops belong to the type H3 of the IUPAC classification. The type H3 hysteresis can be caused by the existence of non-rigid aggregates of plate-like particles or assemblages of slit-shaped mesopores.^28^ The area of the prominent peak in the desorption line reflects the amount of mesopore structure. One can see that it decreased with increasing heating temperature and prolonged heating time. It almost faded away when the temperature reached 400 °C, proving that the slit-shaped mesopores decreased with the increase in heating intensity (higher temperature and longer time). The hysteresis phenomenon of isotherms also existed at low relative pressures to indicate the intercalation, a kind of solid swelling occurring in layered materials. The layer space distance of layered micro-structures in coal expanded with the progress of adsorption so that N_2_ accessed some spaces that were originally unable to allow N_2_ to enter. Nitrogen in the expanded layers hardly escapes from the adsorbent, even if the pressure was low. Low pressure hysteresis indicated that the coal had a layered micro-structure, as will be shown later in CO_2_ adsorption.

For the treatment at 200 °C, the adsorbed N_2_ quantity first increased in the first hour of the heating process and then decreased with increased heating time. Fig. 1(d) shows that the increase in the adsorbed quantity at the beginning of heating process was caused by the increased mesopore volume. In turn, both micropore and mesopore volumes tended to decrease during the continuous thermal treatment. At 300 °C, the adsorption quantity of N_2_ at low pressure (*p*/*p*_0_ < 0.05) sharply decreased, corresponding to the sharp decrease in micropore volume. The continuous decrease of the adsorption quantity at high pressure (*p*/*p*_0_ > 0.05) caused a continuous decrease in mesopore volume. For the heat treatment at 400 °C, the adsorption capacity for N_2_ decreased in the first hour of heating and then increased with heating time. The difference in adsorption capacity was mainly due to micropore adsorption at *p*/*p*_0_ < 0.05. As a result, the mesopore volume diminished first and then remained stable. The micropore volume continuously increased after a sharp decrease in the first hour of heating.

#### 3.1.2 Super-micropores

**Fig. 2:**
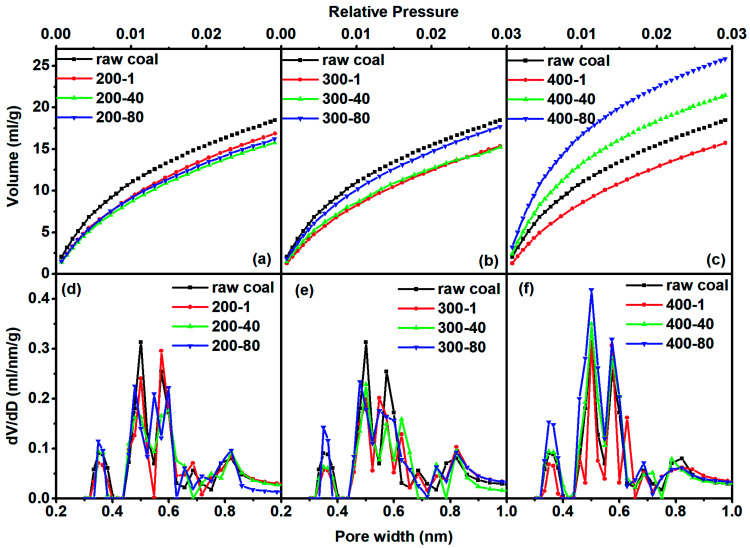
CO_2_ adsorption isotherms [(a)–(c)] and CO_2_-DFT–PSD [(d)–(f)] for different samples.

Fig. 2(a)–(c) show the CO_2_ isotherms of all tested samples, which belong to the type I that represents the characteristics of microporous materials. The PSD obtained with CO_2_ adsorption at 0 °C, in complement to the N_2_ adsorption data, can be used to analyze the narrow microporosity.^27^Fig. 2(d)–(f) display the CO_2_ DFT–PSDs. Intense peaks appeared at 0.35 nm, 0.5 nm, 0.57 nm, 0.68 nm and 0.81 nm. Rajveer, Singh and Rajaura *et al.*^29^ have found the most intense peak at 2*θ* = 26.32° for pristine graphite, indicating an interlayer distance with a spacing of about 0.339 nm determined from the XRD pattern of graphite. Sajjad and Shamaila *et al.*^30^ found that the interlayer spacing is also subject to the degree of oxidation, and the largest interlayer spacing is 0.8133 nm in the graphene oxide. These literature studies proved that the interlayer distance between stacks of aromatic ring structures varies from 0.339 nm to 0.813 nm, as also shown in our measurements. The intense peaks between 0.35 nm and 0.81 nm (0.5 nm, 0.57 nm and 0.68 nm) corresponded to the interlayer distance of stacking aromatic-ring structures at different degrees of oxidation. Consequently, coal has a layered micro-structure, complying well with the hysteresis phenomenon appearing in N_2_ isotherms at low relative pressures. From the data in Fig. 2 we inferred that the super-micropore consisted of inter-layer distances between stacks of aromatic rings.

**Fig. 3:**
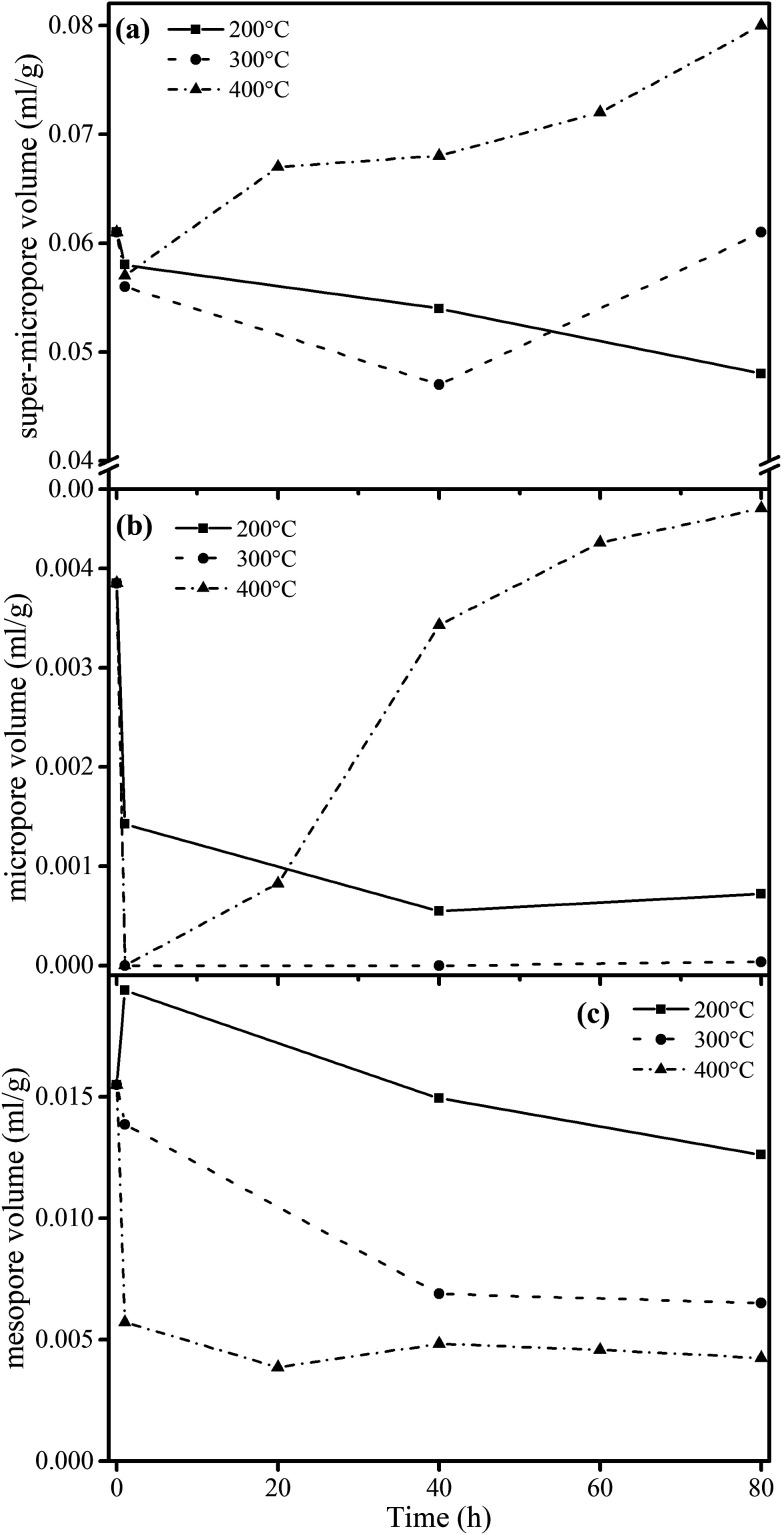
Evolution of pore volume for different samples (calculated by DFT methods) from heat treatments at (a) 200 °C, (b) 300 °C and (c) 400 °C.

For the treatment at 200 °C, the adsorbed CO_2_ quantity continuously decreased as the heating time increased. At 300 °C and 400 °C, the adsorption quantity decreased in the first hour of heating and then increased as the heating time was extended. As a summary, Fig. 3 shows the quantitative analysis data. The evolution of the three types of pore varied with heating temperature with a complex nature. Here we observed the continuous change of pore structure during mild thermal treatment, and the data can be used to inter-connect the evolution characteristics of pore structure and chemical composition of coal, as we will do below.

### 3.2 Variation of chemical composition

**Fig. 4:**
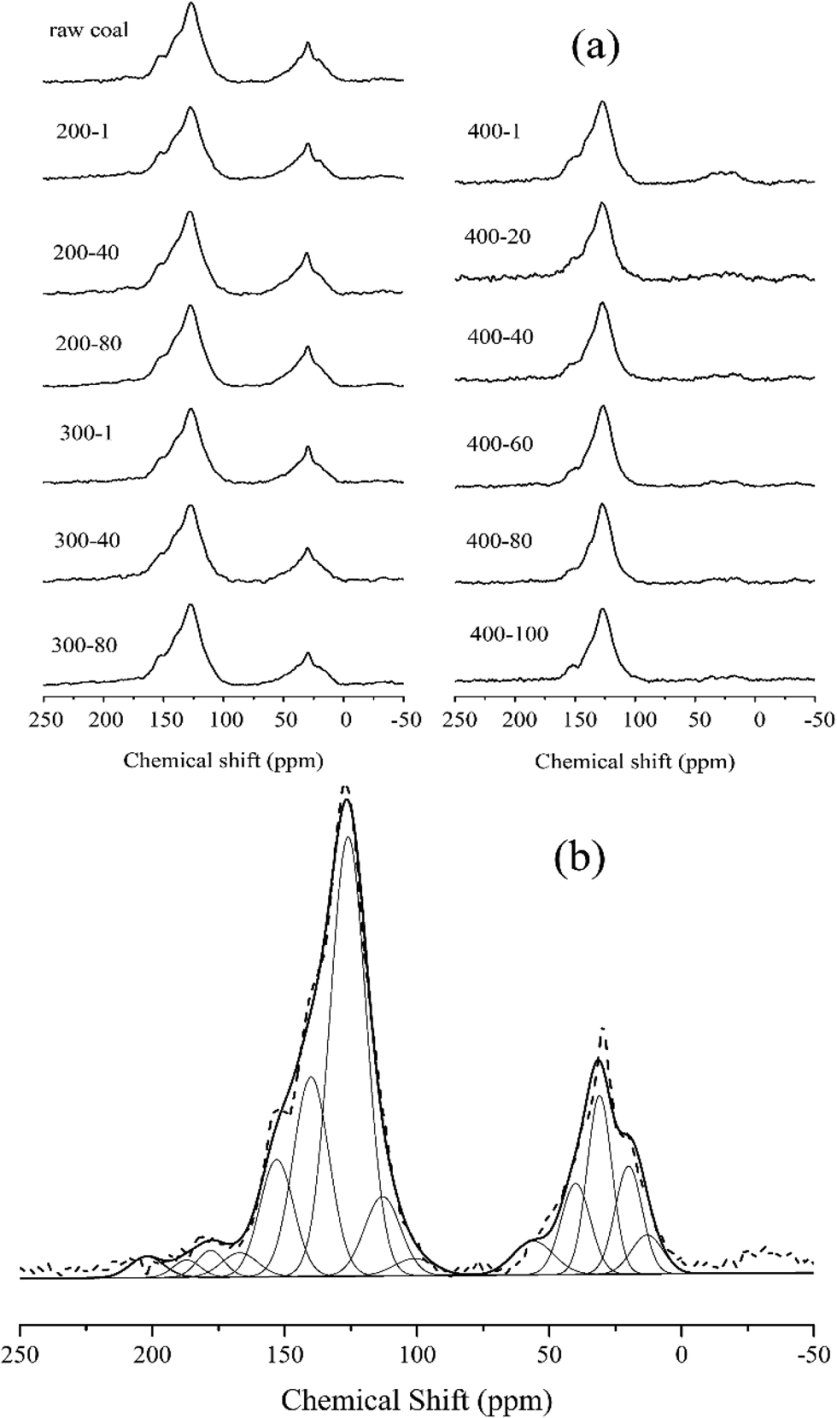
(a) Solid-state ^13^C CP/MAS NMR spectra of different samples and (b) the fitting curve of the spectrum for raw coal.

The ^13^C NMR spectra were taken to quantitatively determine the different types of carbon structure present in coal.^31–33^ We adopted the peak fitting method from Satoru Murata^24^ to separate the ^13^C NMR spectra. Fig. 4(a) shows the ^13^C NMR spectra of 13 samples in total. For the samples of raw coal, 200-1, 200-40, 200-80, 300-1, 300-40 and 300-80, strong bands are seen in the chemical shift regions of 0–98 ppm and 98–202 ppm, which indicate aliphatic and aromatic carbons, respectively. For the samples 400-1 to 400-100 a similar result appears in the aromatic carbon region, and with the progress of reactions the intensity of the peak in the aliphatic carbon region sharply decreased. The result confirmed that the aliphatic carbons are able to escape from the coal matrix at temperatures above 400 °C.

Fig. 4(b) shows the fitting result for raw coal. Assuming that each carbon has an equal sensitivity to magnetic resonance, the amounts of the assigned carbon functional groups could be determined from the relative peak areas based on their carbon atom number. Table 3 shows the fitting results for all samples, with the aromaticity calculated as aromaticity = *A*_98–220_/*A*_t_, where *A*_98–220_ is the area of peaks in the chemical shift region of 98–220 ppm, and *A*_t_ is the total area. The CH_2_/CH_3_ ratio (*R*_CH_2_/CH_3__) in Table 3 reflects the length of aliphatic chains.

**Table 3:** Distribution of carbon functionalities determined by ^13^C NMR (in the ratio of functional group number to 100 carbon atoms)

Sample	Functional groups							Aromaticity	*R*_CH_2_/CH_3__
	Ketone, aldehyde	Carboxylic acid	Ar–O	Ar–C, H	R–O	–CH_2_–	–CH_3_		
Raw coal	2.55	3.50	11.53	55.96	3.98	15.44	7.04	0.73	2.19
200-1	2.34	3.34	11.66	55.53	3.63	15.99	7.51	0.73	2.13
200-40	2.21	2.94	11.65	56.21	4.35	15.51	7.13	0.73	2.17
200-80	1.63	2.82	11.1	57.76	3.63	15.78	7.28	0.73	2.17
300-1	2.26	3.32	10.94	56.16	3.67	15.99	7.66	0.74	2.09
300-40	2.16	3.16	13.02	56.04	4.45	13.71	7.46	0.74	1.84
300-80	1.84	2.64	11.48	60.20	3.51	12.99	7.34	0.76	1.77
400-1	2.23	3.30	12.41	64.55	3.31	7.41	6.79	0.82	1.09
400-20	2.20	3.29	11.78	67.56	4.53	5.27	5.37	0.85	0.98
400-40	2.11	3.10	10.55	71.10	3.92	4.55	4.67	0.87	0.97
400-60	2.01	2.91	9.63	76.01	2.28	3.84	3.32	0.91	1.16
400-80	1.80	2.66	9.04	75.82	2.21	4.30	4.17	0.89	1.03
400-100	1.76	2.24	9.09	75.51	3.26	4.04	4.10	0.89	0.99

When extending the treatment time at 200 °C, the most significant change in the chemical structure was the continuous decrease of the fitting peak areas for the ketone, aldehyde and carboxylic acid functional groups. The aromaticity and CH_2_/CH_3_ ratio are stable. The main chemical variation at 200 °C is thus the decomposition of functional groups such as carboxyl. At 300 °C, the CH_2_/CH_3_ ratio decreased from 2.19 to 1.77 when the treatment time was prolonged to 80 hours. The corresponding aromaticity increased from 0.73 to 0.76. In this heating process, the pyrolysis reaction started. However, the supplied energy was insufficient to cause dramatic reactions. At 400 °C, the CH_2_/CH_3_ ratio decreased from 2.19 (raw coal) to 0.99 for the sample heated for 100 hours (400-100), but the aromaticity conversely increased from 0.73 to 0.89. Compared to the continuous change at 300 °C, the major chemical variation at 400 °C occurred in the first hour of heating, and then there was little variation.

At 200 °C, some carboxyl groups were decomposed to decrease the carboxylic acid peak area in the ^13^C-NMR fitting data. The aromaticity and CH_2_/CH_3_ ratio did not obviously change when prolonging the heating time, indicating that aliphatic hydrocarbons were stable at this temperature. At 300 °C, there was no acute degradation of the vitrinite structure but only a slight change which caused a continuous increase in aromaticity and a decrease in the CH_2_/CH_3_ ratio. When reaching 400 °C, pyrolysis reactions obviously took place to sharply vary the aromaticity and CH_2_/CH_3_ ratio of the sample. Aliphatic side chains and small organic groups should escape from the sample to increase the aromaticity and decrease the CH_2_/CH_3_ ratio.

### 3.3 Relationship between physical and chemical structures

**Fig. 5:**
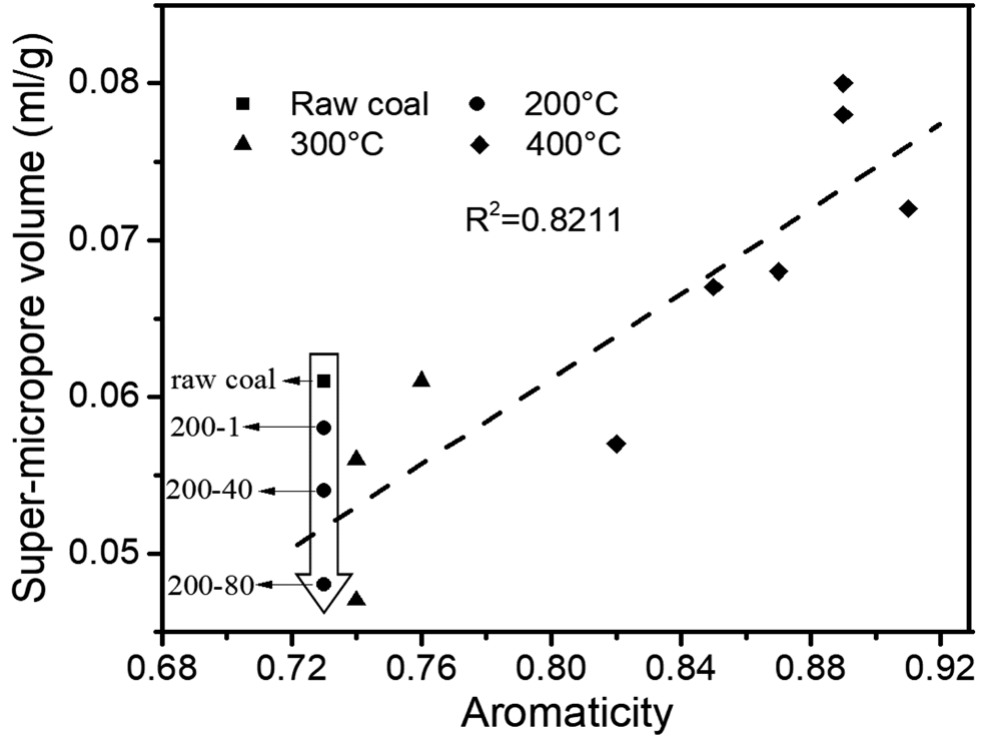
Relationship between aromaticity and super-micropore volume.

**Fig. 6:**
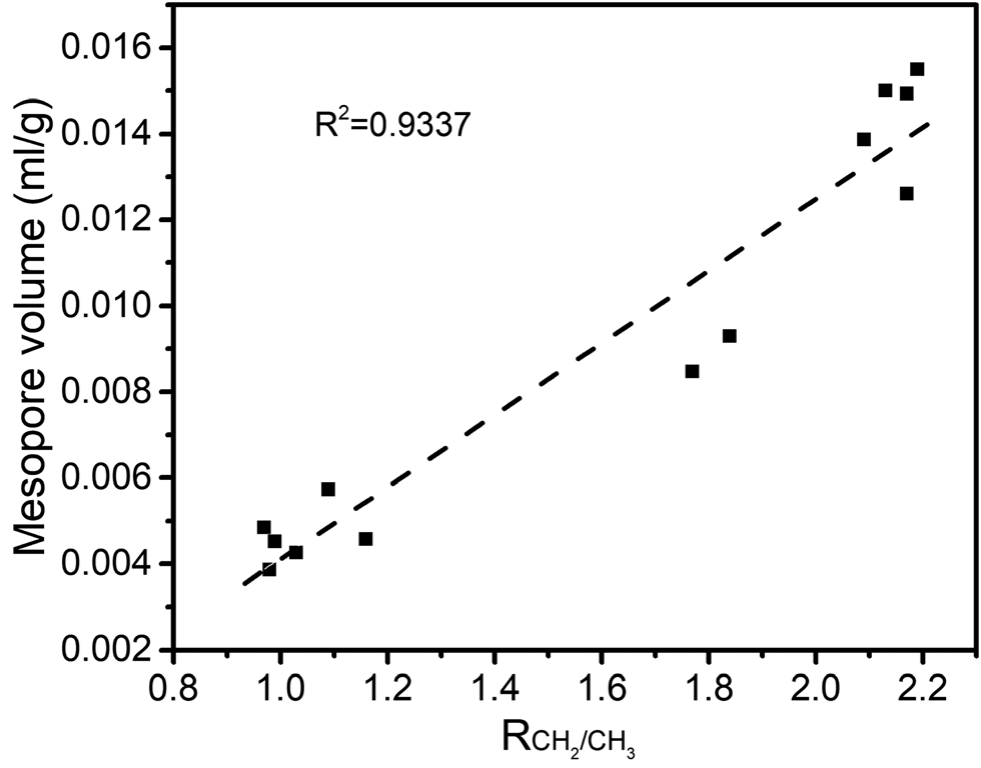
Relationship between *R*_CH_2_/CH_3__ and mesopore volume.

The structure of coal is framed as a three-dimensional cross-linked macromolecular network, with complex and interactive pore and chemical structures.^34^ After quantitative analysis of the pore and chemical structures we adopted line-fitting to reveal the relationship between them. The relationship between the super-micropore volume and aromaticity is shown in Fig. 5. It could be divided into two parts. The first part is for samples with equal aromaticity (raw coal and char prepared at 200 °C) but the super-micropore volume of such a char continuously decreased with prolonged heating time. This occurred mainly because of the decomposition of functional groups such as carboxyl. The thermal treatment at 200 °C thus decreased the interlayer distance of stacking aromatic structures without changing the aromaticity. The second part is for samples with different aromaticities (200-80 and char prepared at 300 and 400 °C) in which the higher the aromaticity, the larger the super-micropore volume. The hysteresis phenomenon of the N_2_-adsorption isotherms at low relative pressures reveals the existence of a layered structure in coal. Furthermore, the CO_2_ adsorption results clarified that the diameter of the super-micropores is equal to the interlayer distances of graphite and graphene oxide. Finally, we confirmed that the layered structure is due to the stacking of aromatic structures by the linear relationship between super-micropores and aromaticity. The relationship between the mesopore volume and the CH_2_/CH_3_ ratio is shown in Fig. 6. The mesopore volume gets larger with a higher CH_2_/CH_3_ ratio. The CH_2_/CH_3_ ratio reflects the length of the aliphatic chains. Therefore, mesopores are formed mainly by the space between macromolecular aromatic rings with aliphatic side chains.

These relationships would promote our understanding of structure evolution during thermal conversion. At 200 °C, the super-micropore volume decreased from 0.061 ml g^−1^ to 0.048 ml g^−1^ without a change in aromaticity, showing that the decreased interlayer space between the stacking aromatic rings is caused by the fracture of hydrogen bonds in oxhydryl and carboxyl species. The micropore pore volume decreased from 0.004 ml g^−1^ to 0.001 ml g^−1^ and the mesopore volume decreased from 0.016 ml g^−1^ to 0.013 ml g^−1^ to show the expansion of the structure frame and the dominant action of heat treatment (no reaction). A slight increase in mesopore volume from raw coal to sample 200-1 at the beginning of the heat treatment should be due to dehydration that released some pores.

Furthermore, at 300 °C, the super-micropore volume decreased from 0.061 ml g^−1^ to 0.047 ml g^−1^ in the first 40 h, indicating the breakage of hydrogen bonds in oxhydryl and carboxyl species (the same as at 200 °C). This volume further increased to 0.061 ml g^−1^ in the later 40 h, possibly due to the increase in aromatic structure as shown in Table 3. The micropore completely disappeared from 0.004 ml g^−1^ in the first hour. In this process the composition of coal slightly changed to decrease the CH_2_/CH_3_ ratio from 2.19 to 1.77 when heating for 80 h, showing essentially the decomposition of aliphatic chains. Finally, the mesopore volume decreased from 0.016 ml g^−1^ to 0.007 ml g^−1^.

With heating at 400 °C, the super-micropore volume decreased from 0.061 ml g^−1^ to 0.051 ml g^−1^ in the first hour, then gradually increased to 0.080 ml g^−1^ in 80 hours and finally remained stable. The increase in aromatic structure is the main reason for this. The micropore volume dropped to zero from 0.004 ml g^−1^ in the first hour and gradually rose again to 0.008 ml g^−1^. At 400 °C there is sufficient energy for volatile compounds to escape from coal to form micropores. The mesopore volume quickly decreased from 0.016 ml g^−1^ to a stable value of 0.006 ml g^−1^ in the first hour. This corresponds with the NMR data showing the decomposition of aliphatic chains and the destruction of mesopores in coal.

## 4. Conclusions

A typical subbituminous coal was heated at a low temperature for a long time to study the evolution of the composition and structure of coal at different temperatures. A quantitative analysis of the chemical structure change was performed in terms of aromaticity and CH_2_/CH_3_ ratio to correlate with the pore structure change of coal in terms of pore size distribution. Treatment at 200 °C caused the sample porosity to continuously decrease with the extension of heating time. At 300 °C, the volumes of mesopores and micropores obviously decreased with the treatment time, and the super-micropore volume also decreased to reach a nadir at 40 hours. When reaching 400 °C, the volume of the micropores and super-micropores decreased first then increased with prolonged heating time, whereas the mesopore volume obviously decreased. Simultaneous studies on chemical structure changes found that at 200 °C, some carboxyl groups decomposed to generate CO_2_. With a heat treatment at 300 °C for a long time, aliphatic chains started to decompose to increase the aromaticity and decrease the CH_2_/CH_3_ ratio. A dramatic pyrolysis took place at 400 °C to cause obvious changes in aromaticity and CH_2_/CH_3_ ratios. The values of aromaticity and the CH_2_/CH_3_ ratio of coal are further found to be proportionally correlative with super-micropore and mesopore volumes, respectively. The super-micropores consist of inter-layer distances between stacks of aromatic rings, and the mesopores are represented by the space between macromolecular aromatic rings which are inter-connected *via* aliphatic chains.

## Conflicts of interest

There are no conflicts of interest to declare.

## Supplementary Material
